# Hidden Giant Third‐Order Nonlinear Circular Dichroism in Chiral Two‐Dimensional Halide Perovskite

**DOI:** 10.1002/advs.78642

**Published:** 2026-11-09

**Authors:** Yuki Takahashi, Daichi Okada, Koshi Oi, Kazuki Mitamura, Takahiko Endo, Yasumitsu Miyata, Taishi Takenobu, Kenichi Yamashita

**Affiliations:** ^1^ Faculty of Electrical Engineering and Electronics Kyoto Institute of Technology Kyoto Japan; ^2^ Department of Applied Physics Graduate School of Engineering Nagoya University Nagoya Aichi Japan; ^3^ Research Center for Materials Nanoarchitectonics National Institute for Materials Science Tsukuba Japan

**Keywords:** anti‐counterfeiting, chirality, nonlinear optical response, third‐harmonic generation, two‐dimensional halide perovskite

## Abstract

Chiral nonlinear optical (NLO) responses provide unique opportunities for manipulating circularly polarized light (CPL) and are attracting increasing interest for advanced photonic and optoelectronic technologies. However, investigations of chiral nonlinear optical materials have largely focused on second‐order processes, while the role of chirality in third‐order NLO responses, particularly in third‐harmonic generation (THG), remains largely unexplored. Here, we demonstrate hidden giant THG circular dichroism (THG‐CD) in the chiral two‐dimensional halide perovskite (R‐MBACl)_2_PbI_4_ through polarization‐resolved THG measurements, and further examine the THG‐CD in several structurally different Pb‐based two‐dimensional perovskites. Under excitonic resonant conditions, incident right‐ and left‐handed CPL generates nearly orthogonally polarized THG emission, resulting in a reversal of the THG‐CD response between the two orthogonal output polarization components. Consequently, nearly perfect CPL discrimination is achieved under polarization‐resolved detection, with dissymmetry factors exceeding |*g*_*THG* − *CD*_| = 1.9. In contrast, these chiral NLO responses are largely canceled in conventional polarization‐integrated THG measurements, resulting in apparently weak circular dichroism and masking the existence of giant chiral optical responses. These unique optical responses establish a versatile platform for polarization‐encoded photonic technologies, including circularly polarized light detection, optical information encryption, and anti‐counterfeiting systems operating at technologically relevant telecommunication wavelengths.

## Graphical abstract

We demonstrate hidden giant third‐harmonic generation circular dichroism (THG‐CD) in chiral two‐dimensional halide perovskite. Although conventional polarization‐integrated THG measurement shows almost no chiral optical response, polarization‐resolved measurement reveals a hidden capability for nearly perfect discrimination of incident circularly polarized light helicity. This finding opens new opportunities for polarization encoded chiral optical application such as encryption and anti‐counterfeiting system.

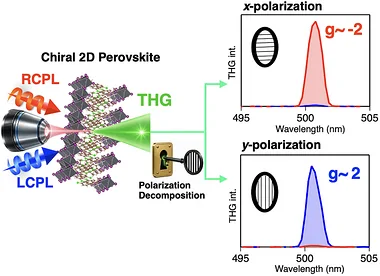

## 1 Introduction

Discrimination of the handedness of chiral information, such as circularly polarized light (CPL) and spin‐polarized electrons, provides an additional degree of freedom for optoelectronic systems and enables advanced information processing. Chiral materials offer an ideal platform for such functionalities, as their intrinsic structural chirality induces anisotropic interactions with chiral information carriers [1, 2, 3, 4, 5]. For chiral light discrimination, the most fundamental manifestation is circular dichroism (CD), which arises from differential absorption of left‐ and right‐handed CPL. In most materials, however, the magnitude of CD is typically far less than one percent, which severely limits its practical applicability. For practical applications, more efficient approaches for the discrimination of CPL are therefore required.

Among the various optoelectronic phenomena, nonlinear optical (NLO) responses and photocurrent generation are widely recognized as powerful properties for CPL discrimination. Upon irradiation or excitation with CPL, the NLO signal [6, 7, 8, 9, 10, 11, 12] or generated photocurrent [13, 14, 15, 16, 17, 18] can exhibit pronounced handedness dependence, with the difference reaching several tens of percent. Such remarkable chiroptical sensitivity has fueled intense interest in exploration of novel chiral materials or system for achievement further better sensitivity. In particular, chiral organic–inorganic hybrid materials provide a versatile platform that combines the structural tunability of organic molecules with the strong light–matter interactions of low‐dimensional inorganic semiconducting frameworks. This synergy gives rise to the distinctive chirality‐related phenomena and opens new opportunities for superior optoelectronic applications [19, 20, 21, 22, 23].

In the field of chiral nonlinear optics, a wide variety of organic‐inorganic hybrid materials have been explored, leading to remarkable advances in CPL discrimination. In some cases, handedness discrimination, quantified by dissymmetry factors approaching unity, has been achieved [24]. To date, however, the majority of studies have primarily focused on second‐order NLO processes, in which chirality‐induced symmetry breaking plays a key role in nonlinear chiroptical responses that can be probed through discrimination or generation of CPL, such as second harmonic generation circular dichroism (SHG‐CD) [6, 7, 8, 9, 10, 11, 12] and circularly polarized second harmonic generation (CP‐SHG) [25, 26, 27, 28, 29]. In contrast, the contribution of chirality to third‐order nonlinear frequency conversion process like third harmonic generation (THG) has not yet been systematically investigated, despite the fact that organic–inorganic metal halides are known to exhibit strong third‐order nonlinear optical responses owing to their pronounced excitonic properties arising from their low‐dimensional structures [30, 31, 32, 33, 34, 35]. To date, studies of chiral THG have been largely limited to chiral metamaterial‐based systems, including metallic and semiconducting helicoidal colloids [36, 37, 38, 39, 40, 41, 42, 43, 44]. The role of chirality in THG responses in organic and organic–inorganic hybrid materials, however, remains largely unexplored.

In this study, we investigate THG circular dichroism (THG‐CD) in the chiral two‐dimensional (2D) halide perovskite (R/S‐MBACl)_2_PbI_4_. The material exhibits pronounced handedness‐dependent THG responses, enabling efficient discrimination of CPL and wavelength‐converted detection of telecom‐band information into the visible region, which is not achievable with conventional reported SHG‐CD process. Polarization‐resolved measurements further reveal that the orthogonally polarized THG components possess oppositely signed THG‐CD signals, which largely cancel out under conventional unpolarized total‐THG detection. Remarkably, the handedness of the incident CPL is directly converted into highly orthogonal linearly polarized THG emission. By separating these polarization components with an analyzer, a giant hidden chiral NLO response with dissymmetry factors exceeding |*g*_THG‐CD_| > 1.9 is uncovered. Such a unique chiral response is further exploited to demonstrate a novel chiral anti‐counterfeiting system. These findings establish THG‐CD as a powerful platform for chiral information processing and reveal previously hidden chiral functionalities for advanced optical technologies.

## 2 Results and Discussion

Chiral two‐dimensional (2D) halide perovskites, (R/S‐MBACl)_2_PbI_4_, were mainly used in this study (Figure S1). This material has been extensively investigated previously, from fundamental chiroptical properties to chiral SHG, where pronounced anisotropic SHG‐CD responses have been reported [25, 45, 46]. The NLO responses of single crystal (R/S‐SC) and thin‐film (R/S‐TF) of (R/S‐MBACl)_2_PbI_4_ were examined (Section S2), with the primary focus placed on the R/S‐SC samples. Owing to their strong excitonic character, the R/S‐TF exhibit intense and spectrally sharp excitonic absorption (Figure 1b). Such an excitonic character is beneficial for third‐order NLO response.

**FIGURE 1:**
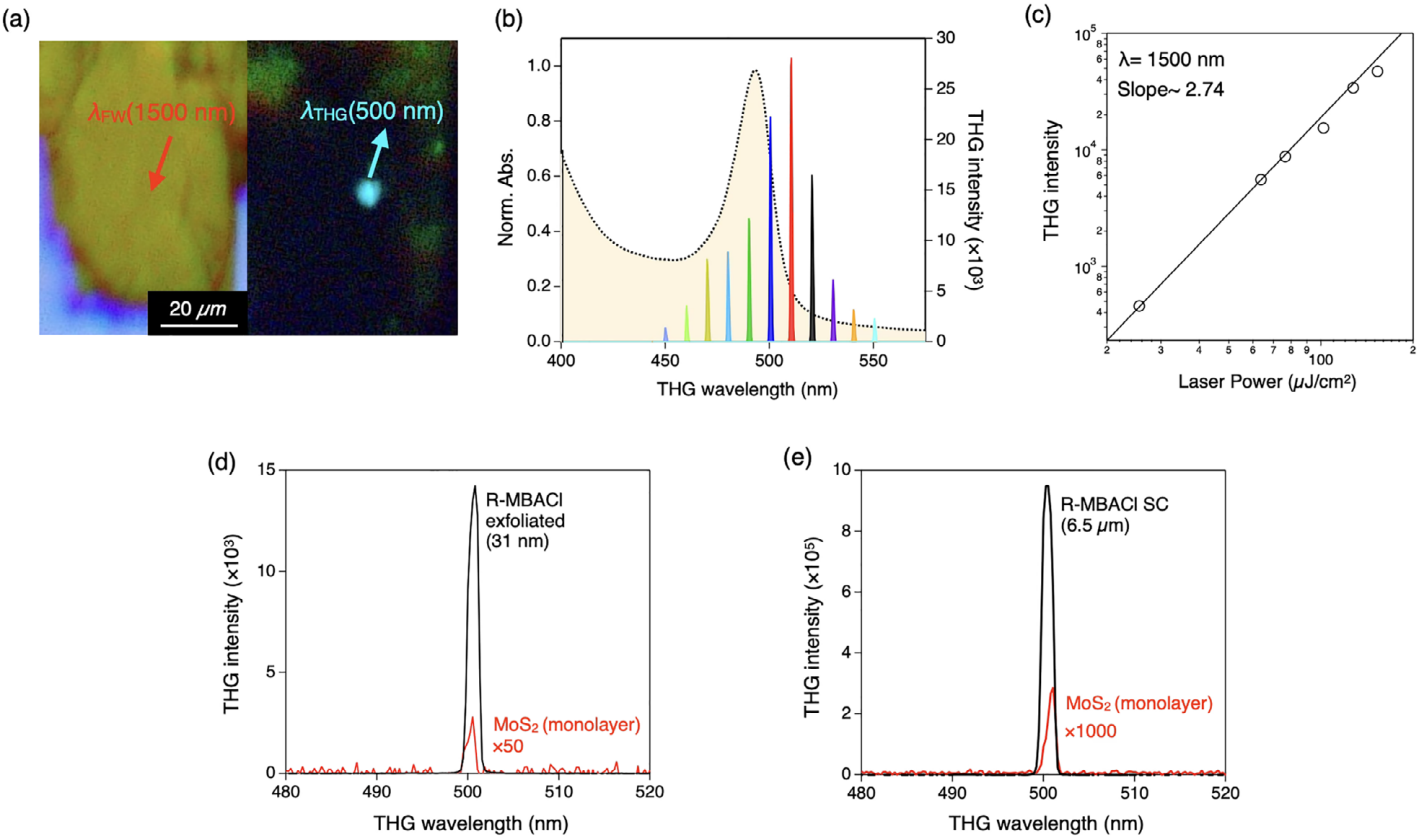
(a) Optical microscopy image of the crystal under 1500 nm laser irradiation, showing green emission at 500 nm generated via THG. (b) THG spectra measured under incident laser wavelengths ranging from 1450 to 1650 nm at a constant laser power of 200 µJ/cm^2^ and the normalized absorption spectrum of R‐thin film. The measured bulk crystalline thickness is 6.5 µm. (c) THG intensity as a function of incident laser power at *λ* = 1500 nm. The solid line represents a power‐law fit, showing the third‐order nonlinear optical nature. (d,e) Comparison of THG spectra at *λ*_THG_ = 500 nm between thin exfoliated (31 nm) (d) or bulk R‐SC (6.5 µm) sample (e) and MoS_2_ monolayer.

THG measurement was conducted using a home‐built optical setup (Section S3 and Figure S2). The samples were irradiated using a wavelength‐tunable picosecond laser source, and the generated harmonic signals were collected using a spectrometer equipped with a CCD. The chiral perovskite crystal was rotated in‐plane and positioned so that the incident linear polarization was aligned with the crystalline axis corresponding to the maximum THG intensity. Under irradiations at 1500 nm, the R/S‐SC exhibited clear frequency up‐conversion into the visible color (Figure 1a). By scanning the incident laser wavelength from 1450 to 1650 nm, clear sharp peaks corresponding to the third‐harmonic signals were observed at each incident wavelength (Figure 1b). In addition, the signal intensity exhibited a nearly cubic (fitted slope∼2.74) dependence on the incident laser power (Figure 1c). These data provide clear evidence of THG. The THG efficiency reached its maximum at approximately 510 nm (Figure 1b), slightly red‐shifted from the excitonic absorption peak at 495 nm. This redshift is likely attributed to the interplay between the excitonic resonance enhancement of THG and self‐absorption in the relatively thick single crystal.

The THG conversion efficiency of (R/S‐MBACl)_2_PbI_4_ was investigated by comparing with CVD‐grown monolayer MoS_2_ [47]. The effective NLO susceptibility of MoS_2_ has been reported previously, therefore, it serves as a useful reference material. Because of the substantial difference in thickness between the bulk crystal and the monolayer material, a direct comparison between them cannot be considered entirely fair. Therefore, the bulk SC was exfoliated and prepared thin SC sample. By comparing the THG responses of the 31 nm thin R‐SC with that of monolayer MoS_2_ (Figure 1d), the effective third‐order NLO susceptibility at λ_THG_ = 500 nm was evaluated. After considering the absorption coefficient, phase mismatch, refractive index, and sample thickness [33, 48, 49, 50], the effective NLO susceptibility of the R‐SC at an excitation wavelength of 1500 nm was estimated to be approximately 0.1 times that of monolayer MoS_2_ (Section S4). Based on the reported data for monolayer MoS_2_ at 1560 nm [49], its effective susceptibility at 1500 nm was estimated and used to obtain an effective susceptibility of 10.4 × 10^−29^ *m* ^2^/*V* ^2^ for (R‐MBACl)_2_PbI_4_. Although the THG efficiency is lower than that of monolayer MoS_2_, its large macroscopic interaction length enables significantly stronger THG generation in the bulk crystalline state. For example, 6.5 µm thick R‐SC crystal exhibits a THG intensity several thousand times greater than that of monolayer MoS_2_ (Figure 1e).

Next, anisotropic response to incident circular polarization was investigated. By setting the quarter waveplate (QWP) at ± 45° with respect to the incident polarization direction, we can choose a L‐ or R‐CP handedness of fundamental light. Figure 2a shows the normalized THG spectra measured from selected R‐ and S‐SC samples under CPL irradiation in the incident wavelength range of 1440–1560 nm, showing a clear anisotropy in the THG intensity. Figure 2b plots the wavelength dependence of the THG dissymmetry factor (*g*‐factor), which is defined as *g*_THG − CD_ =  2(*I_L_* − *I_R_*)/(*I_L_* + *I_R_*), where *I_L_* and *I_R_* refers to the output THG intensity for the fundamental L‐ or R‐CPL, respectively. In both R‐ and S‐SC crystals, clear chiral light discrimination in the third order NLO response was observed throughout the measured NIR range. Although variations in the g‐factor were observed among samples, these differences are likely attributable to variations in crystal quality and thickness. The power dependence of the THG intensity under CPL excitation was also investigated using the same crystal as that measured in Figure 1c. For both L‐ and R‐CPL, the slopes of the power dependence were nearly identical to each other and to that obtained under linearly polarized excitation, indicating that the *g*‐factor is almost independent of the incident laser power (Figure 2c). Figure 2d summarizes previously reported SHG‐CD *g*‐factors measured at fundamental wavelengths longer than 1000 nm [51, 52, 53, 54, 55, 56] and compares them with the highest *g*‐factors obtained for R/S‐SC in Figure 2b. Among them, the longest reported CPL detection wavelength was 1200 nm in spiral [(R/S)‐MBA]_4_Bi_2_Br_10_ microcrystals, which exhibited a *g*_THG − CD_ of 0.58 [52]. In contrast, the present chiral 2D halide crystals enable CPL discrimination at much longer wavelengths extending into the optical communication band, while converting these long‐wavelength photons into the visible region through the THG process.

**FIGURE 2:**
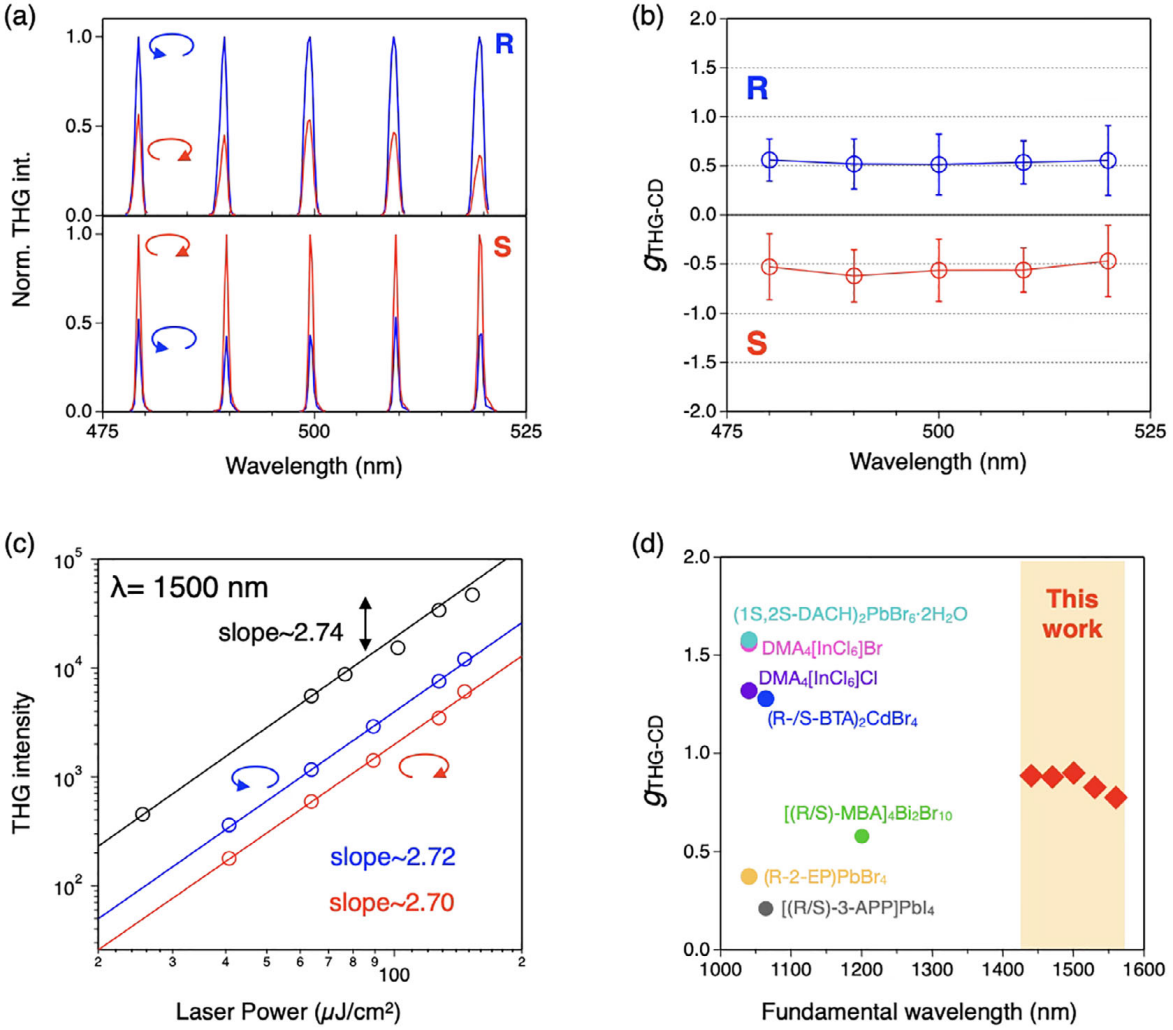
(a) Normalized THG spectra under right‐ and left‐circularly polarized irradiation for the R‐ and S‐SC samples, showing anisotropic handedness‐dependent responses. (b) Wavelength dependence of g_THG‐CD_ for the R‐ and S‐SC samples. Data represent the average g_THG‐CD_ values obtained from 4 independently measured SC sample; error bars show the standard deviation between these samples. (c) THG intensity as a function of incident laser power under RCP (red) and LCP (blue) irradiation at *λ*_THG_ = 500 nm for the same R‐SC sample as in Figure 1c. The slope obtained under linear polarization in Figure 1c is also shown as a black dot and line for comparison. (d) Comparison of the *g*_THG‐CD_ values obtained in this work with previously reported *g*_SHG‐CD_ values measured at incident wavelengths longer than 1000 nm [51, 52, 53, 54, 55, 56].

To further investigate the detailed chiral optical response of our samples, QWP rotation analysis of the output THG was performed. In this measurement, the incident polarization state was continuously changed by rotating the QWP, while the generated THG signal was resolved into its *x*‐ and *y*‐polarized components. Here, the *x* ‐axis was defined as the in‐plane direction exhibiting the maximum THG intensity under linearly polarized irradiation, with the orthogonal direction defined as the *y*‐axis. The THG signal was analyzed along these two directions. Further details of the measurement geometry and polarization configuration are provided in Section S3 (Figure S2). Unless otherwise noted, all data presented hereafter were obtained using an incident wavelength of 1500 nm. At first, we selected a crystal exhibiting a good THG‐CD response and investigated its QWP rotation angle dependence. Figure 3a,d shows the THG intensity as a function of the QWP angle for the total THG signal detected without a polarizer. Figure 3b,e shows the corresponding *x*‐ and *y*‐polarized THG components, and their normalized profiles are presented in Figure 3c,f. The conventional *g*_THG − CD_ values, defined by the total THG intensity, were estimated to be 0.70 and −0.80 for the R‐ and S‐SC samples, respectively (Figure 3a,d). However, interestingly, when the THG signal is decomposed into *x*‐ and *y*‐polarized components, while the *x*‐polarization is dominant for total THG, the chiral response of each polarization component is found to be clearly reversed (Figure 3b,c,e,f). For the R‐SC (S‐SC) sample, the *x*‐polarized THG component exhibits a pronounced minimum, approaching nearly zero intensity, at QWP angles corresponding to R‐CPL (L‐CPL), while the *y*‐polarized component almost reaches to its maximum under the same CPL direction. These results also indicate that the handedness of incident CPL is encoded into the orthogonal linear polarization of THG (Figure S3). For example, in the case of R‐SC, incident R‐CPL generates THG with an *x*‐polarization purity of up to 99%, whereas L‐CPL excitation predominantly produces y‐polarized THG with a polarization purity of 77% (Figure S3). Therefore, exceptionally large dissymmetry factors are obtained in such polarization‐resolved condition. For the R‐SC sample, *g*_*x*, THG − CD_ =   − 1.66 and *g*_*y*, THG − CD_ =  1.95 were obtained for the *x*‐ and *y*‐polarized components, respectively. In contrast, the signs were completely reversed for the S‐SC sample, yielding *g*_*x*, THG − CD_ =  1.77 and *g*_*y*, THG − CD_ =   − 1.40. These findings reveal that a giant chiral response is hidden within the polarization‐resolved THG components. Since the chiral contributions of the *x*‐ and *y*‐polarized THG signals exhibit opposite signs, they compensate each other in the total signal. Thus, conventional *g*‐factor analyses based on the total NLO signal underestimate the intrinsic chiral response.

**FIGURE 3:**
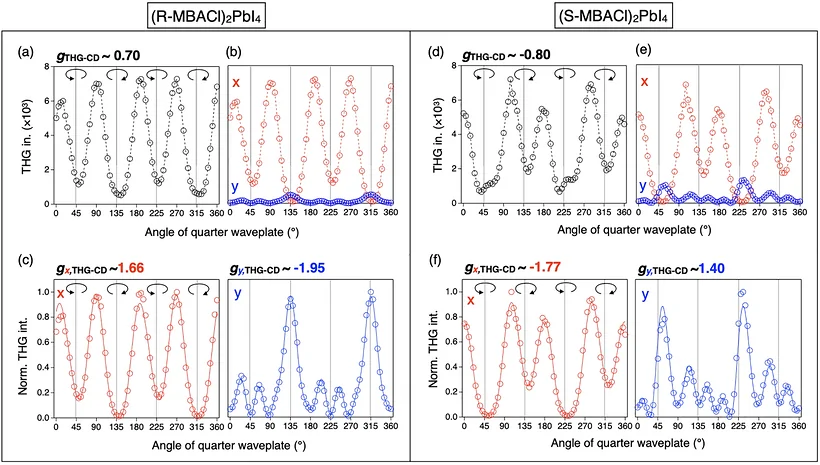
(a,d) Total‐THG intensity detected without polarizer as a function of QWP rotation angle for R‐ (a) and S‐SC (d) sample under the irradiation of 1500 nm incident wavelength. (b,e) x‐ (red) and y‐polarized (blue) THG intensity profile as a function of QWP rotation angle for R‐ (b) and S‐SC (e) sample. (c,f) Normalized THG intensity profiles for the *x*‐ and *y*‐polarized components obtained from Figure 3b,e. The solid line shows the fitting curve evaluated by Equation (4). The obtained fitting value is expressed in Table S1.

The (R/S‐MBACl)_2_PbI_4_ SC belong to triclinic *P*1 structure [45], which possesses the lowest crystal symmetry. Owing to this low‐symmetry crystal structure, all components of the third‐order nonlinear susceptibility tensor remain nonzero and independent. Under the present experimental configuration employing normal incidence and normal detection, the nonlinear polarization can be expressed using the susceptibility tensor components as follows (Section S5) [57]:

(1) $$\left(\begin{array}{c} P_{X}^{\left(3\omega \right)}\\ P_{Y}^{\left(3\omega \right)} \end{array}\right)=\frac{\varepsilon_{0}}{4}\;\left(\begin{array}{cccc} \chi_{11} & \chi_{12} & \chi_{18} & \chi_{19}\\ \chi_{21} & \chi_{22} & \chi_{28} & \chi_{29} \end{array}\right)\left(\begin{array}{c} E_{X}^{3}\\ E_{Y}^{3}\\ 3E_{X}E_{Y}^{2}\\ 3E_{X}^{2}E_{Y} \end{array}\right)$$

For CPL dependent NLO response, considering the relation  *E_x_* =   ± *iE_y_*, the asymmetric response to opposite circular polarizations originates from the $E_{y}^{3}$ and $E_{x}^{2}E_{y}$ terms. Accordingly, the tensor components χ_12_, χ_19_, χ_22_, and χ_29_ are responsible for generating the circularly anisotropic response. The THG intensity difference between CPL handedness (Δ*I*(3ω)_*THG* − *CD*_) can be expressed as:

(2) $$\begin{array}{cc} & \Delta I{\left(3\omega \right)}_{THG-CD}\propto 4Im\left[\left(\chi_{11}-3\chi_{18}\right){\left(\chi_{12}-3\chi_{19}\right)}^{*}\right]\\ & \;\times \left(x-polarization\right) \end{array}$$

(3) $$\begin{array}{cc} & \Delta I{\left(3\omega \right)}_{THG-CD}\propto 4Im\left[\left(\chi_{21}-3\chi_{28}\right){\left(\chi_{22}-3\chi_{29}\right)}^{*}\right]\\ & \;\times \left(y-polarization\right) \end{array}$$

These expressions indicate that the chiral response originates from the phase difference between tensor components exhibiting symmetric responses to CPL (χ_11_, χ_18_, χ_21_, and χ_28_), and those responsible for asymmetric responses (χ_12_, χ_19_, χ_22_, and χ_29_). Importantly, in the P1 crystal structure, the x‐ and y‐polarized THG components are governed by different sets of independent tensor elements. As a result, the relative phase relationships between the effective symmetric and asymmetric tensor contributions can differ for the two polarization channels, allowing the THG‐CD response to exhibit different signs and magnitudes. Accordingly, the opposite signs of the chiral response observed for the x‐ and y‐polarized THG components indicate a reversal of the effective phase relationship between the corresponding tensor contributions.

A detailed determination of the individual third‐order tensor amplitudes and phases is challenging because the QWP‐angle‐dependent THG response involves complex interference among multiple susceptibility components. Therefore, the angular dependence of the THG intensities was fitted as a function of the waveplate rotation angle φ using a Fourier series of harmonic functions (Figure 3c,f; see Section S5):

(4) $$\begin{array}{cc} I\;\left(3\omega \right) & =A+B\cos2\varphi +C\cos4\varphi +D\cos6\varphi +E\cos8\varphi \\ & \;+F\cos10\varphi +G\cos12\varphi +H\sin2\varphi +I\sin4\varphi \\ & \;+J\sin6\varphi +K\sin8\varphi +L\sin10\varphi +M\sin12\varphi \end{array}$$

Here, the coefficients are expressed as complicated linear combinations of the nonlinear susceptibility tensor components. The obtained fitting coefficients are summarized in Table S1. Using the same QWP angular reference for all measurements, the sign reversal associated with chiral inversion is predominantly observed in the coefficients of the sine terms, indicating that the enantiomer‐sensitive response is primarily reflected in the odd components of the angular dependence under this angular system.

As shown in Figure 2b, the *g*_ THG − CD_ evaluated from the total THG intensity exhibits some deviation among different crystals. Considering that differences in crystal thickness may contribute to this variation, we further performed QWP rotation analysis on several crystals with different thicknesses to examine whether the pronounced polarization‐dependent chiral optical response is preserved (Figure S4). Although the magnitude of *g*_ THG − CD_ varied among crystals, all samples exhibited giant chiral optical responses with opposite signs in the orthogonal polarization components, consistent with the results shown in Figure 3. The opposite chiral responses in the two polarization components partially cancel each other, with the degree of cancellation varying among crystals and leading to differences in the observed total  *g*_ THG − CD_. Importantly, the consistently observed giant polarization‐resolved THG‐CD means that the 2D perovskite investigated here intrinsically possesses NLO tensor components with relative amplitudes and phases that give rise to such giant chiral responses, as discussed later. The observed variations in the dissymmetry factor may be associated with thickness‐dependent propagation effects, such as birefringence‐induced phase accumulation of the fundamental light and phase mismatch during THG generation, which can modify the effective relative contributions of these tensor components. Interestingly, this cancellation between the orthogonal polarization resulted in a nearly vanishing *g*_ THG − CD_ in some crystals. As shown in Figure 4a, one such crystal exhibited almost no detectable THG‐CD in the total THG intensity (*g*_ THG − CD_ =  0.04). However, polarization‐resolved analysis revealed pronounced THG‐CD responses with opposite signs in the *x*‐ and *y*‐polarized components, with  *g*_*x*,*THG* − *CD*_ =  1.93 and *g*_*y*,*THG* − *CD*_ =   − 1.92, respectively, demonstrating nearly perfect discrimination between L‐ and R‐CP fundamental irradiations (Figure 4a,b). These results highlight the importance of polarization‐resolved measurements, which enable the extraction of chiral responses that may remain hidden in conventional THG‐CD measurements. At the same time, the present system offers a unique chiral functionality in which the sign of a giant nonlinear circular dichroism can be switched simply by selecting the output polarization with a polarizer without changing the chiral enantiomeric structure (Figure 4e). In addition, the emitted THG exhibits predominantly linear polarization, with up to 97% of the total THG intensity detected in the *x*‐ and *y*‐polarized components under L‐CPL and R‐CPL excitation, respectively, allowing the incident CPL helicity to be directly identified from the polarization state of the emitted THG (Figure S5). This was further confirmed by Stokes parameter analysis (*S*_1_, *S*_2_, and *S*_3_), which revealed that the two output THG polarization states were nearly linear, with their polarization azimuths differing by approximately 93°, which clearly means orthogonal polarization states (Section S9 and Figure S6). Such a polarization resolved giant *g*‐factor was also confirmed in the S‐SC sample exhibiting a weak *g*_ THG − CD_ response, as well as across the incident wavelength range of 1440–1560 nm (Figures S7 and S8a).

**FIGURE 4:**
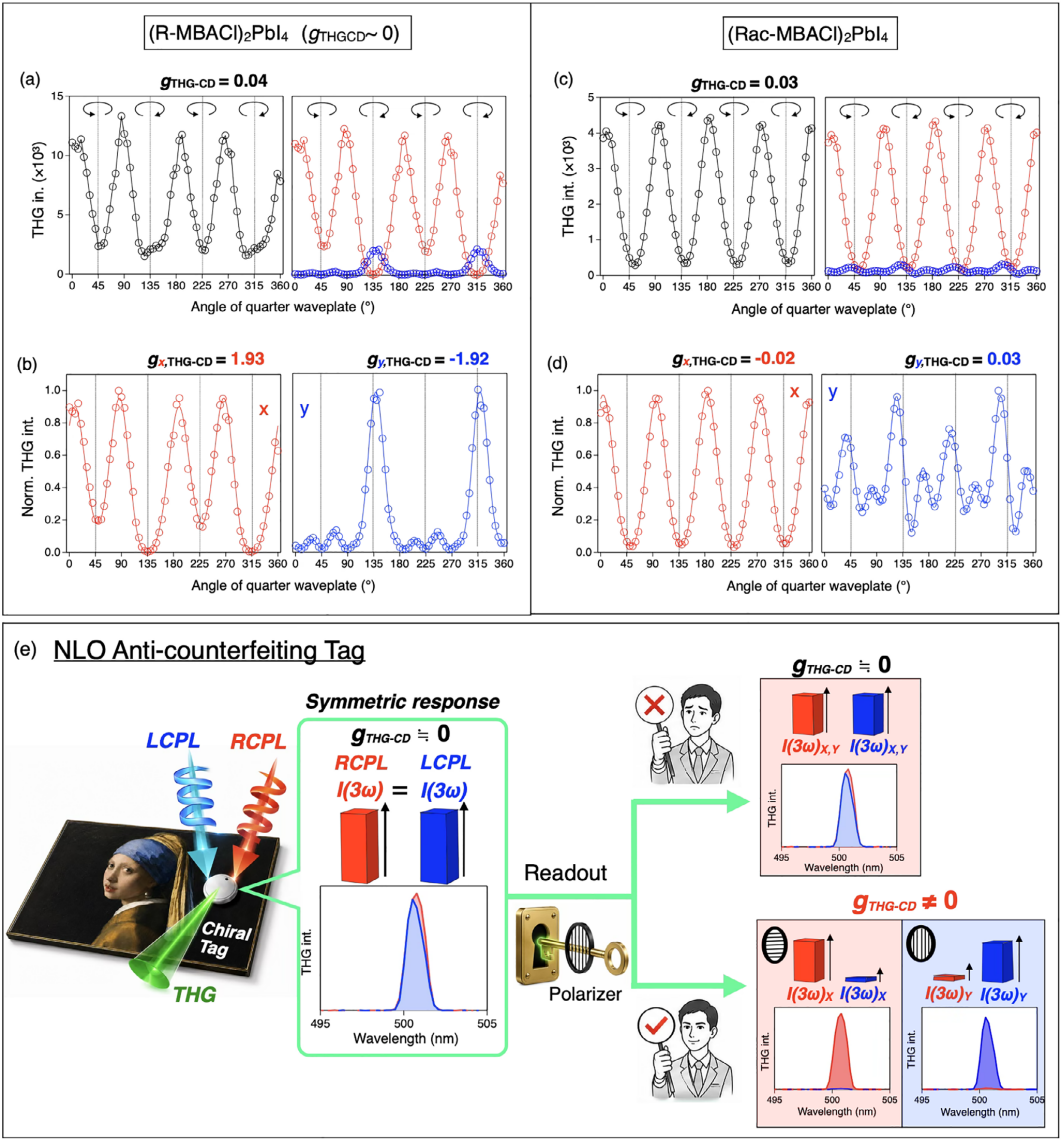
(a,c) THG intensity as a function of QWP rotation angle for R‐ (a) and Rac‐SC (b) sample. The left panel showing black dot is total THG intensity profile and right panel show x‐ red dot) and y‐polarization (blue dot) resolved THG profile data. R‐SC data show a case in which the overall g‐factor is nearly negligible, whereas giant chiral responses emerge upon polarization‐resolved analysis. (b,d) Normalized THG intensity profiles for the x‐ and y‐polarized components obtained from the right panels of Figure 4a,c. The solid line shows the fitting curve evaluated by Equation 4. (e) The schematic representation of the concept for potential application to anti‐counterfeiting systems. The hidden information can only be accessible through a polarizer.

A detailed determination of the third‐order tensor amplitudes and phases is difficult because the complex nature of the third‐order nonlinear susceptibility makes the analysis of chiral THG highly complicated. Nevertheless, the polarization‐resolved THG‐CD response, with ∣*g*_THG − CD_∣ approaching its theoretical maximum of 2, allows us to infer a possible relationship between the relative amplitudes and phases of the contributing tensor components. According to Equations (2) and (3), the maximum value of *g*_THG − CD_ is achieved when the symmetric and chiral asymmetric tensor contributions possess equal amplitudes, i.e., ∣χ_11_ − 3χ_18_∣  =  ∣χ_12_ − 3χ_19_∣ and ∣χ_21_ − 3χ_28_∣  =  ∣χ_22_ − 3χ_29_∣, together with a relative phase difference of  ΔΦ  =   ± π/2 (Section S5). Therefore, the giant THG‐CD responses observed here suggest that these interference conditions closely satisfied in the present crystal sample, which is further supported by the observed polarization conversion from incident CPL to orthogonally polarized THG (Section S5).

Notably, this pronounced anisotropic THG‐CD response was observed only under resonant conditions near the excitonic transition (Figure S8b). In general, nonlinear susceptibilities can exhibit pronounced amplitude and phase variations near electronic resonances because of their complex dispersive nature, whereas such phase variations are typically less pronounced far from resonance. The observed spectral correlation therefore suggests that the excitonic resonance may contribute to establishing the effective amplitude and phase relationships required for the large THG‐CD response. However, since the present measurements do not directly determine the spectral evolution of the complex tensor components, the excitonic resonance is considered here as a possible factor facilitating these amplitude and phase conditions, rather than as their definitive origin.

In addition, we investigated the THG responses of racemic (Rac)‐SC samples and an achiral reference compound, (PEA)_2_PbI_4_, which crystallizes in the $P\bar{1}$ space group [58, 59]. In both crystalline systems, *g*‐factor remained nearly zero or very small not only in the total THG intensity but also under polarization‐resolved conditions (Figure 4c,d and Figure S9). Furthermore, the THG activity itself under CPL irradiation was much weaker than that observed in the chiral SC samples. These results indicate that chirality contributes not only to the generation of anisotropic chiral THG responses but also to the enhancement of light‐matter interactions with CPL. Such a clear distinction between chiral and racemic (achiral) crystals, particularly in the presence or absence of giant hidden chiral responses, opens up a new design concept for NLO anti‐counterfeiting technologies (Figure 4e). In the original state, the anti‐counterfeiting tag simply acts as an infrared‐to‐visible wavelength conversion material and exhibits equivalent responses to opposite CPL, making the hidden information inaccessible. However, for authentic tags, polarization‐resolved detection reveals hidden chiral responses that become observable only after introducing an analyzer, whereas counterfeit tags remain optically inactive toward CPL even under identical polarization‐resolved conditions. Such selective emergence of chiral responses enables reliable authentication based on hidden NLO signatures (Figure 4e). Moreover, the polarization‐dependent inversion of the chiral NLO response provides an additional degree of freedom for authentication. In addition to the presence or absence of hidden chiral responses, the sign reversal of the chiral NLO state induced by switching the output polarization can serve as an extra authentication parameter. Such multiplexed optical information is expected to further enhance the security level and anti‐counterfeiting performance of the proposed tag system (Figure 4e).

Finally, as additional information, although we have so far discussed the THG‐CD response mainly based on (R/S‐MBACl)_2_PbI_4_, we also investigated other chiral 2D perovskites with different chiral crystal structures. Specifically, we examined (R‐MBA)_2_PbI_4_ belonging to the chiral P2_1_2_1_2_1_ space group [60] and (R‐3BrMBA)_2_PbI_4_ belonging to the chiral P2_1_ space group [61]. Interestingly, similar hidden giant THG‐CD behavior was also observed in both crystals (Section S13 and Figure S10). These results suggest that the observed polarization‐dependent THG‐CD is not specific to (R/S‐MBACl)_2_PbI_4_ or its triclinic *P*1 structure. These observations are also consistent with the tensor nature of the THG response. Although the higher symmetries of the P2_1_ and P2_1_2_1_2_1_ structures impose more restrictions on the allowed tensor components than the P1 structure, multiple third‐order nonlinear susceptibility tensor contributions can still be independently involved in each output polarization channel. Consequently, the *x*‐ and *y*‐polarized THG signals can reflect different effective combinations of these tensor contributions. Therefore, the effect of structural chirality can be manifested differently in the two polarization channels, possibly resulting in THG‐CD responses with different magnitudes and opposite chiral optical signs.

## 3 Conclusion

In summary, we demonstrated efficient chiral third‐order NLO responses in chiral 2D halide perovskites, arising from the combination of strong excitonic effects and pronounced chiral light–matter interactions. These characteristics enable efficient CPL discrimination through the THG process at technologically important optical communication wavelengths. Beyond establishing chiral 2D perovskites as a promising platform for THG‐based CPL discrimination, our study uncovers a hidden chiral optical effect, whereby oppositely signed chiral responses residing in orthogonally polarized THG channels are largely concealed through cancellation in the total THG signal. By selectively accessing polarization‐resolved channels, giant chiral responses can be revealed. Importantly, similar behavior was observed in several chiral 2D perovskites with different crystal structures, suggesting that this hidden chiral response is not specific to a single material or crystal symmetry. These findings highlight that the apparent weakness of a chiroptical response does not necessarily reflect the strength of the underlying chiroptical interactions, providing a valuable evaluation framework for uncovering hidden and enhanced chiral optical responses in future material systems. Through efficient CPL discrimination beyond the spectral range of SHG and the introduction of an additional degree of freedom via polarization encoding, this THG‐based platform broadens the design space of chiral nonlinear optics and paves the way for future applications in anti‐counterfeiting, secure optical communications, and advanced information processing.

## Author Contributions

**Yuki Takahashi**: data curation, investigation, validation, formal analysis. **Daichi Okada**: conceptualization, methodology, funding acquisition, formal analysis, validation, supervision, writing – original draft, data curation, visualization, project administration. **Koshi Oi**: resources. **Kazuki Mitamura**: resources. **Takahiko Endo**: resources. **Yasumitsu Miyata**: funding acquisition, resources, writing – review and editing. **Taishi Takenobu**: funding acquisition, resources, writing – review and editing. **Kenichi Yamashita**: funding acquisition, supervision, writing – review and editing, project administration.

## Funding

This work was supported by the Grants‐in‐Aid for Scientific Research (JP22H00215, JP22H04957, JP 23H01942, JP24KK0084, JP24H00044, JP25H00741, JP25K22242, JP25H00835, JP26K01475) from the Japan Society for the Promotion of Science (JSPS) and JST CREST (JPMJCR20T4, JPMJCR23A4, JPMJCR23O3) and JST FOREST (JPMJFR213X) from Japan Science and Technology Agency (JST).

## Conflicts of Interest

The authors declare no conflicts of interest.

## Supporting information




**Supporting File**: advs78642‐sup‐0001‐SuppMat.pdf

## Data Availability

The data that support the findings of this study are available from the corresponding author upon reasonable request.
