# Supplementary material for: Hidden Giant Third‐Order Nonlinear Circular Dichroism in Chiral Two‐Dimensional Halide Perovskite
Source: Adv Sci (Weinh). 2026 Nov 9:e78642. Online ahead of print. doi: 10.1002/advs.78642 (PMC13749012; doi:10.1002/advs.78642)
Supplement: Supplementary file 1 [file ADVS-9999-e78642-s001.pdf]

Supporting Information

Hidden Giant Third-Order Nonlinear Circular Dichroism in Chiral Two-dimensional Halide Perovskite

Yuki Takahashi [1], Daichi Okada [1], Koshi Oi[2], Kazuki Mitamura[2], Takahiko Endo[3], Yasumitsu Miyata[3], Taishi Takenobu [2] and Kenichi Yamashita[1]*

[1,] *Faculty of Electrical Engineering and Electronics, Kyoto Institute of Technology, Matsugasaki, Sakyo-ku, Kyoto, 606-8585, Japan*

[2,] *Department of Applied Physics, Graduate School of Engineering, Nagoya University, Furo-cho, Chikusa-ku, Nagoya, Aichi 464-8603, Japan*

[3,] *Research Center for Materials Nanoarchitectonics, National Institute for Materials Science, Tsukuba, 305-0044, Japan*

* To whom correspondence should be addressed.
E-mail: oka-d@kit.ac.jp

1. Materials

Unless otherwise noted, all reagents and solvents were used as received. (R)-4-Chloro-α-methylbenzylamine (R-MBACl) is purchased from Thermo scientific. (S)-4-Chloro-α-methylbenzylamine (S-MBACl) is purchased from Apollo scientific. 57% w/w HI aqueous solution , 2-phenylehtylamine (PEA) and (R/S)-methylbenzylamine were purchased from TCI. 50% H_3PO_2 aqueous solution is purchased from Sigma-aldrich. PbO was purchased from Nacalai tesque.

2. Sample preparation (Single crystal, thin-film)

For the synthesis of $(R/S\text{-}MBACl)_2PbI_4$, $(PEA)_2PbI_4$, $(R\text{-}3BrMBA)_2PbI_4$ and $(R\text{-}MBA)_2PbI_4$ single crystals, 400 mg of PbO powder and 400 μL of the corresponding chiral ammonium precursor were dissolved in 15 mL HI solution with 1 mL of H_3PO_2 as a stabilizer at 150°C until all solid was completely dissolved and a transparent yellow solution was formed. The solution was then slowly cooled to room temperature (25°C) over 48 hours, during which single crystals of 2D-OIHPs gradually precipitated. The obtained crystals were filtered, washed with toluene, and dried under vacuum for more than 48 hours. In case of synthesis for $(racemic\text{-}MBACl)_2PbI_4$, R and S-MBACl were mixed in a 1:1 ratio (200 μL: 200 μL).

The $(R/S\text{-}MBACl)_2PbI_4$ thin film was prepared by spin-coating onto a quartz glass substrate The synthesized single crystals were dissolved in DMF at a concentration and the thin film was obtained by spin-coating the solution for 60 seconds, followed by annealing at 80°C for 15 minutes. To encapsulate the sample, 100 nm PMMA film was also spin-coated on top of the 2D-OIHPs film.

The MoS_2 monolayer used as the THG reference was synthesized by exfoliation and CVD growth. The CVD-grown MoS_2 monolayer was fabricated as described in Ref. [47].

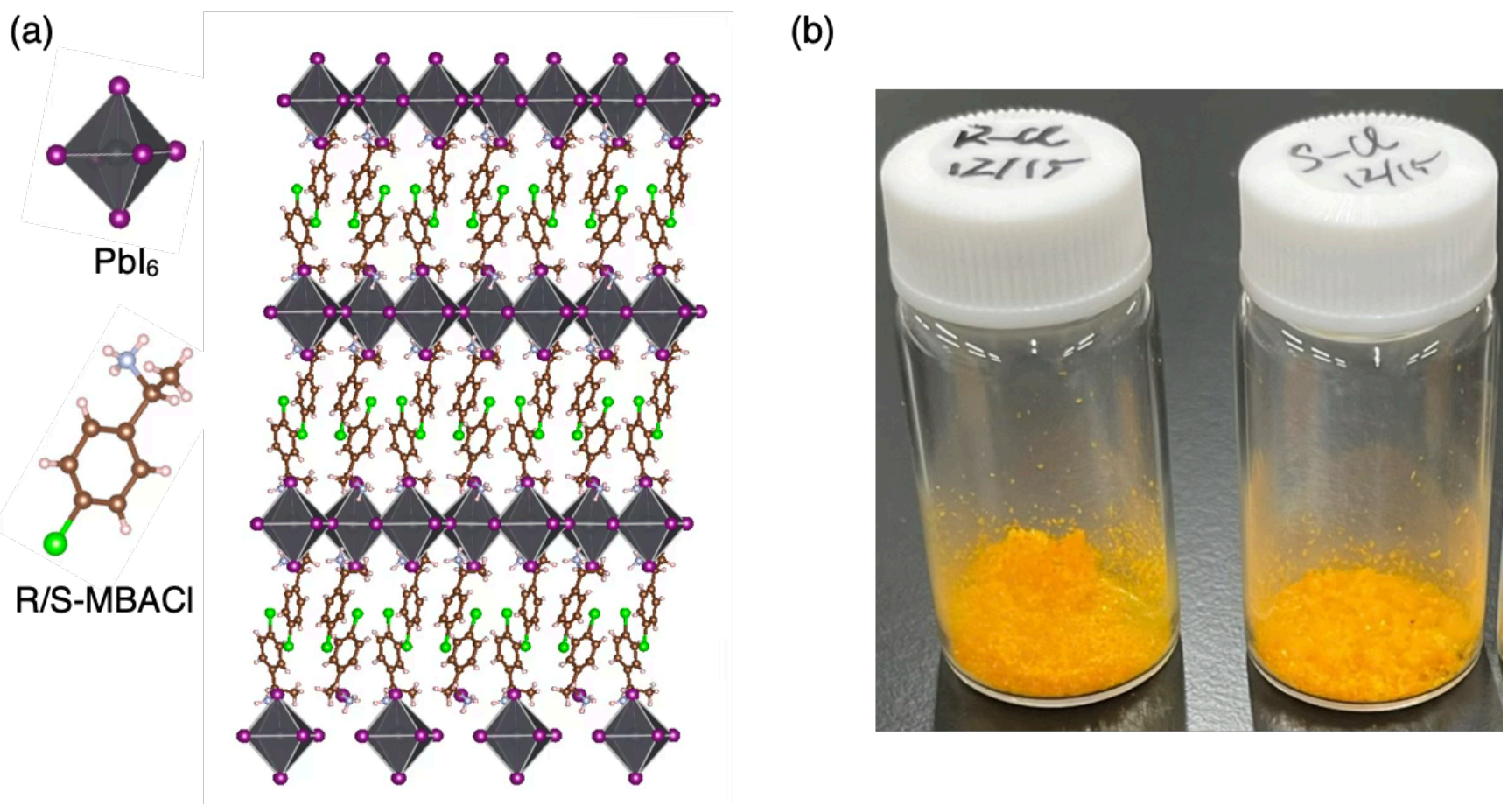

Figure S1. (a) Crystalline structure of $(R/S\text{-}MBACl)_2PbI_4$ single crystal, which is reported in reference [45]. (b) Photograph of synthesized $(R/S\text{-}MBACl)_2PbI_4$ crystals.

3. Optical setup for THG measurement

THG was measured by home-made optical setup as shown in Figure S2. A pico-second pulsed laser system (EKSPLA: PT403, 410 ~2300 nm) with a pulse width ~15 *ps* and a repetition rate of 1 kHz was used for fundamental incident light source. Incident laser polarization was fixed *x*-polarization by polarizer and intensity is modulated by tunable ND filter. The laser beam was focused to a spot size of ~20 μm using a plano-convex lens. The THG signal was collected by an EMCCD camera (Andor Newton) through a spectrometer (Andor Kymera 193i). For the quarter waveplate (QWP) rotational measurements, a QWP was placed before the sample to generate circularly polarized light, and an additional polarizer was set before the spectrometer to analyze the polarization state of the output THG signal. Before the THG measurements, the sample was rotated so that the in-plane direction exhibiting the maximum THG intensity under linearly polarized excitation was aligned with the incident x-polarization.

For the quarter-wave plate (QWP) rotation measurements, a QWP was placed before the sample to continuously vary the polarization state of the incident fundamental light, and an additional polarizer was placed before the spectrometer as an analyzer to resolve the polarization of the output THG signal. Before the THG measurements, the sample was rotated such that the in-plane direction exhibiting the maximum THG intensity under linearly polarized excitation was aligned parallel to the incident linear polarization, and this direction was defined as the *x* axis. The orthogonal in-plane direction was defined as the *y*-axis. At a QWP rotation angle of 0°, the fast axis of the QWP was aligned parallel to the *x*-axis. For polarization-resolved THG measurements, the analyzer was oriented parallel to either the *x* or *y* axis to selectively detect the corresponding THG component. The coordinate system and polarization configuration are schematically illustrated in Figure S2.

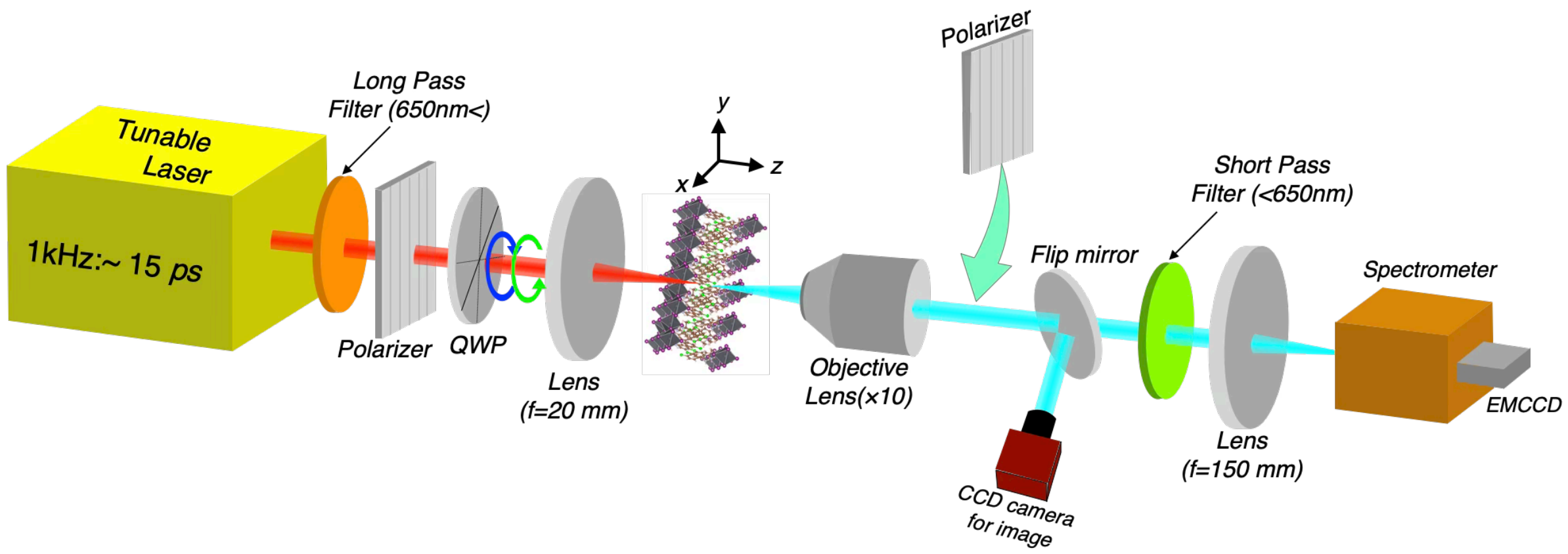

Figure S2. Schematic representation of optical set up. Here, *z*-direction is defined as laser propagation direction.

4. Comparison with MoS_2

In order to compare the effective third-order NLO susceptibility between $(R\text{-}MBACl)_2PbI_4$ and MoS_2, the following equation is used. [33]

$$\frac{\chi^{(3)}_{R-SC}}{\chi^{(3)}_{MoS2}} = \left[\frac{(I_3 n_3 {n_1}^3/{\omega_3}^2)_{R-SC}}{(I_3 n_3 {n_1}^3/{\omega_3}^2)_{MoS2}}\left(\frac{\left({\Delta k_{(3)}}^2 + {a_3}^2/4\right)e^{a_3 L}}{e^{-a_3 L} - 2e^{-a_3 L/2}\cos\left(\Delta k_{(3)} L\right) + 1}\right)_{R-SC}\left(\frac{e^{-a_3 L} - 2e^{-a_3 L/2}\cos\left(\Delta k_{(3)} L\right) + 1}{\left({\Delta k_{(3)}}^2 + {a_3}^2/4\right)e^{a_3 L}}\right)_{MoS2}\right]^{\frac{1}{2}}$$

where I_3 is the THG intensity; n_3 and n_1 are the refractive index at the THG and fundamental wavelengths, respectively; ω_3 is the angular frequency of the THG; α_3 is the absorption coefficient at the THG wavelength; and $\Delta k_{(3)}$ is the wavevector mismatch between the THG wave and the fundamental wave. The refractive index of the 2D perovskite and the refractive index and absorption coefficient of MoS_2 were taken from the literature. [48-50]

The effective third-order NLO susceptibility of MoS_2 has been reported $(1.7 \pm 0.6) \times 10^{-28}\ m^3/V^2$ at 1560 nm.[49] Thus, at first we estimated NLO susceptibility of MoS_2 at 1500 nm and compared it with perovskite samples.

5. THG analysis

THG-CD analysis for single crystal

The third-order nonlinear optical susceptibility tensor can be expressed in matrix form by allowing permutations of the Cartesian coordinates x, y, and z.

$$\chi^{(3)} = \begin{pmatrix} \chi_{11} & \chi_{12} & \chi_{13} & \chi_{14} & \chi_{15} & \chi_{16} & \chi_{17} & \chi_{18} & \chi_{19} & \chi_{1,10} \\ \chi_{21} & \chi_{22} & \chi_{23} & \chi_{24} & \chi_{25} & \chi_{26} & \chi_{27} & \chi_{28} & \chi_{29} & \chi_{2,10} \\ \chi_{31} & \chi_{32} & \chi_{33} & \chi_{34} & \chi_{35} & \chi_{36} & \chi_{37} & \chi_{38} & \chi_{39} & \chi_{3,10} \end{pmatrix}$$

$$\begin{matrix} xxx & yyy & zzz & yzz & yyz & xzz & xxz & xyy & xxy & xyz \\ 1 & 2 & 3 & 4 & 5 & 6 & 7 & 8 & 9 & 10 \end{matrix}$$

$(R/S\text{-}MBACl)_2PbI_4$ single crystals belong to triclinic $P1$ space group. Owing to the low crystal symmetry, all third-order nonlinear susceptibility tensor components are allowed and independent.

In this study, the z-axis is defined as the laser propagation direction. Under normal incidence and normal detection, the tensor components associated with the z-direction vanish, and the susceptibility tensor can be simplified as follows:

$$\chi^{(3)} = \begin{pmatrix} \chi_{11} & \chi_{12} & 0 & 0 & 0 & 0 & 0 & \chi_{18} & \chi_{19} & 0 \\ \chi_{21} & \chi_{22} & 0 & 0 & 0 & 0 & 0 & \chi_{28} & \chi_{29} & 0 \\ 0 & 0 & 0 & 0 & 0 & 0 & 0 & 0 & 0 & 0 \end{pmatrix}$$

The third-order nonlinear polarization is described as follows;

$$\begin{pmatrix} P_X^{(3\omega)} \\ P_Y^{(3\omega)} \\ P_Z^{(3\omega)} \end{pmatrix} = \frac{\varepsilon_0}{4} \begin{pmatrix} \chi_{11} & \chi_{12} & 0 & 0 & 0 & 0 & 0 & \chi_{18} & \chi_{19} & 0 \\ \chi_{21} & \chi_{22} & 0 & 0 & 0 & 0 & 0 & \chi_{28} & \chi_{29} & 0 \\ 0 & 0 & 0 & 0 & 0 & 0 & 0 & 0 & 0 & 0 \end{pmatrix} \begin{pmatrix} E_X^3 \\ E_Y^3 \\ E_Z^3 \\ 3E_Y E_Z^2 \\ 3E_Y^2 E_Z \\ 3E_X E_Z^2 \\ 3E_X^2 E_Z \\ 3E_X E_Y^2 \\ 3E_X^2 E_Y \\ 6E_X E_Y E_Z \end{pmatrix}$$

$$= \frac{\varepsilon_0}{4} \begin{pmatrix} \chi_{11} & \chi_{12} & \chi_{18} & \chi_{19} \\ \chi_{21} & \chi_{22} & \chi_{28} & \chi_{29} \end{pmatrix} \begin{pmatrix} E_X^3 \\ E_Y^3 \\ 3E_X E_Y^2 \\ 3E_X^2 E_Y \end{pmatrix}$$

Thus, the THG intensities corresponding to the *x*- and *y*-polarized components are expressed as follows, respectively:

$I(3\omega) \propto \left[\frac{\epsilon_0}{4}(\chi_{11}E_X^3 + \chi_{12}E_Y^3 + 3\chi_{18}E_X E_Y^2 + 3\chi_{19}E_X^2 E_Y)\right]^2$ (*x*-polarization)

$I(3\omega) \propto \left[\frac{\epsilon_0}{4}(\chi_{21}E_X^3 + \chi_{22}E_Y^3 + 3\chi_{28}E_X E_Y^2 + 3\chi_{29}E_X^2 E_Y)\right]^2$ (*y*-polarization)

The total THG intensity is given by the sum of both polarization components.

THG-CD is defined as the intensity difference under fundamental circularly polarized irradiation. The circular polarization states are expressed as

$$E_y = \pm i E_X$$

First, it is clear that the electric field components responsible for the asymmetric THG-CD response under circularly polarized excitation are E_y^3 and $E_x^2 E_y$. Accordingly, the existence of the associated tensor components is required for the emergence of THG-CD. For the triclinic $P1$ structure, these tensor components correspond to χ_{12}, χ_{19}, χ_{22}, and χ_{29}.

Substituting $E_y = iE_x$ and $E_y = -iE_x$ into the THG intensity expressions and taking their difference, $\Delta I(3\omega)_{THG-CD}$, yields the THG-CD response:

$\Delta I(3\omega)_x \propto 4Im[(\chi_{11} - 3\chi_{18})(\chi_{12} - 3\chi_{19})^*]$ (x-polarization)

$\Delta I(3\omega)_y \propto 4Im[(\chi_{21} - 3\chi_{28})(\chi_{22} - 3\chi_{29})^*]$ (y-polarization)

The phase difference among the nonlinear susceptibility tensor components gives rise to the anisotropic THG-CD response. Although the triclinic *P*1 structure allows all tensor components to be independent, making it difficult to quantitatively evaluate the contribution of each tensor component individually, the sign reversal of the *g*-factor between the *x*- and *y*-polarized components in (R/S-MBACl)$_2$PbI$_4$ single crystals clearly indicates an inversion of the phase relationship among the tensor components contributing to the THG-CD response. This behavior suggests that the tensor components governing the *x*- and *y*-polarized THG responses perceive the structural crystal chirality differently, resulting in opposite chiral NLO responses in the two polarization components.

In this study, the g-factor is defined as $g_{THG-CD} = 2\,(I_L - I_R)/(I_L + I_R)$, where I_L and I_R refers to the to the output THG intensity for the fundamental L- or R-CPL, respectively. By expressing $(\chi_{11} - 3\chi_{18}) = Ae^{i\Phi_A}$ and $(\chi_{12} - 3\chi_{19}) = Be^{i\Phi_B}$, the g-factor is expressed as follow;

$$g_{x\ or\ y,THG-CD} = \frac{4|A||B|\sin(\phi_A - \phi_B)}{|A|^2 + |B|^2}$$

The above equation indicates that a giant THG-CD response ($\left|g_{x\ or\ y,THG-CD} \sim 2\right|$) can be realized when the symmetric and asymmetric tensor contributions possess comparable amplitudes and a relative phase difference close to $\pm\frac{\pi}{2}$. Specifically, the interference term $Im[(\chi_{11} - 3\chi_{18})(\chi_{12} - 3\chi_{19})^*]$ becomes maximal when $(\phi_A - \phi_B) = \pm\frac{\pi}{2}$, while the additional condition $|A| = |B|$ is required to achieve the theoretical limit of the g-factor. Therefore, the experimentally observed giant g-factor implies an almost ideal interference condition between the contributing tensor components.

Such a near-ideal interference condition is also evidenced by the polarization conversion from incident CPL to orthogonally polarized THG, because $(\chi_{11} - 3\chi_{18}) = i(\chi_{12} - 3\chi_{19})$ or $(\chi_{21} - 3\chi_{28}) = i(\chi_{22} - 3\chi_{29})$ conditions lead to the near extinction of one THG linear polarization component.

<u>Fitting for THG intensity as function of QWP rotation angle</u>

When the rotation angle of the quarter-wave plate (QWP) is defined as ϕ, the electric field components E_x and E_y after the QWP can be expressed using the Jones matrix formalism as

$$\begin{pmatrix} E_x \\ E_y \end{pmatrix} = E_0 \begin{pmatrix} cos^2\varphi + isin^2\varphi \\ \sin\varphi\cos\varphi\,(1 - i) \end{pmatrix}$$

The fitting analysis was performed using polarization-resolved THG intensities. As described above, the x-and y-polarized THG intensities are expressed as

$$I(3\omega) \propto \left[\frac{\epsilon_0}{4}(\chi_{11}E_X^3 + \chi_{12}E_Y^3 + 3\chi_{18}E_XE_Y^2 + 3\chi_{19}E_X^2E_Y)\right]^2 \text{ (x-polarization)}$$

$$I(3\omega) \propto \left[\frac{\epsilon_0}{4}(\chi_{21}E_X^3 + \chi_{22}E_Y^3 + 3\chi_{28}E_XE_Y^2 + 3\chi_{29}E_X^2E_Y)\right]^2 \text{ (y-polarization)}$$

Expanding the electric field terms as functions of the QWP rotation angle ϕ yields

$$E_x{}^3 = E_0{}^3[(cos^6\varphi - 3cos^2\varphi sin^4\varphi) + i(3cos^4\varphi sin^2\varphi - sin^6\varphi)]$$

$$E_y{}^3 = -2E_0{}^3 sin^3\varphi cos^3\varphi(1 + i)$$

$$E_x E_y{}^2 = E_0{}^3[2sin^4\varphi cos^2\varphi - 2isin^2\varphi cos^4\varphi]$$

$$E_x{}^2 E_y$$
$$= E_0{}^3 sin\varphi cos\varphi[(cos^4\varphi - sin^4\varphi + 2cos^2\varphi sin^2\varphi)$$
$$+ i(-cos^4\varphi + sin^4\varphi + 2cos^2\varphi sin^2\varphi)]$$

As shown above, the third-order electric-field terms, $E_x{}^3$, $E_y{}^3$, $E_x E_y{}^2$, and $E_x{}^2 E_y$, already contain complex and many higher-order angular dependences on the QWP rotation angle φ. The THG intensity is obtained from the squared modulus of the nonlinear polarization, in which these complex electric-field terms are combined with the third-order susceptibility tensor components. When the susceptibility tensor components are also treated as complex quantities, the resulting intensity expression contains numerous interference terms involving both their amplitudes and relative phases, making a detailed determination of the individual tensor components difficult. Therefore, rather than attempting a full tensor decomposition, we analyze the QWP-angle-dependent THG intensity using a Fourier series of harmonic functions as follows;

$$I(3\omega)$$
$$= A + B\cos 2\varphi + C\cos 4\varphi + D\cos 6\varphi + E\cos 8\varphi + F\cos 10\varphi + G\cos 12\varphi$$
$$+ H\sin 2\varphi + I\sin 4\varphi + J\sin 6\varphi + K\sin 8\varphi + L\sin 10\varphi + M\sin 12\varphi$$

where the coefficients A-M are represented by complicated linear combinations of nonlinear susceptibility tensor components.

The fitting value obtained in Figure 3c, d is summarized following table.

	A	B	C	D	E	F	G	H	I	J	K	L	M
R-x	0.462	-0.023	0.406	-0.044	0.043	0	0	0.140	0.109	0.055	0	0	0
S-x	0.420	0	0.265	0.066	0.013	0	0	-0.233	0.170	-0.087	0.024	0	0
R-y	0.281	0.011	-0.222	0	-0.012	0	0	-0.258	0.028	0.151	0.015	-0.067	0
S-y	0.270	-0.137	-0.141	0.155	-0.0468	-0.033	0	0.122	-0.100	-0.083	0.094	0.046	0

Table S1. The fitting value obtained in Figure 3c, d

6. Linear polarization degree of THG under the CPL irradiation in Figure 3a, b

Here, the degree of linear polarization was defined as stokes parameter, $S_1 = (I_x - I_y)/(I_x + I_y)$.

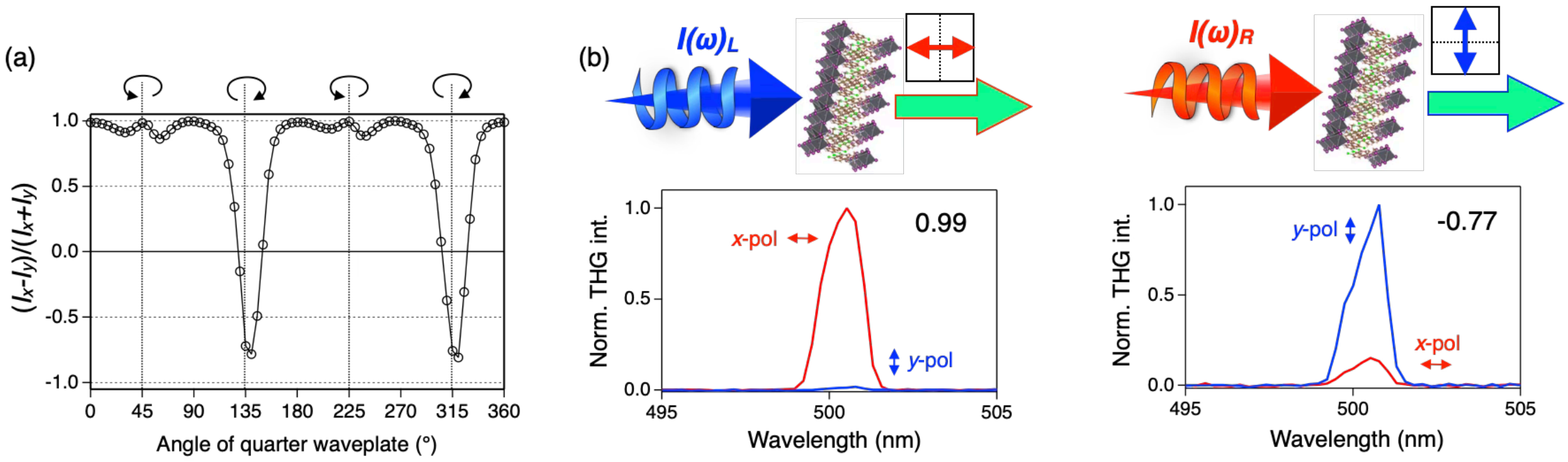

Figure S3. (a) Evaluated linear polarization degree as S_1 value of stokes parameter as function of QWP angle. (b) Extracted normalized *x*- and *y*-polarized THG spectrum under left and right CPL irradiation.

7. Polarization resolved QWP rotation analysis of R-SC samples with different thickness

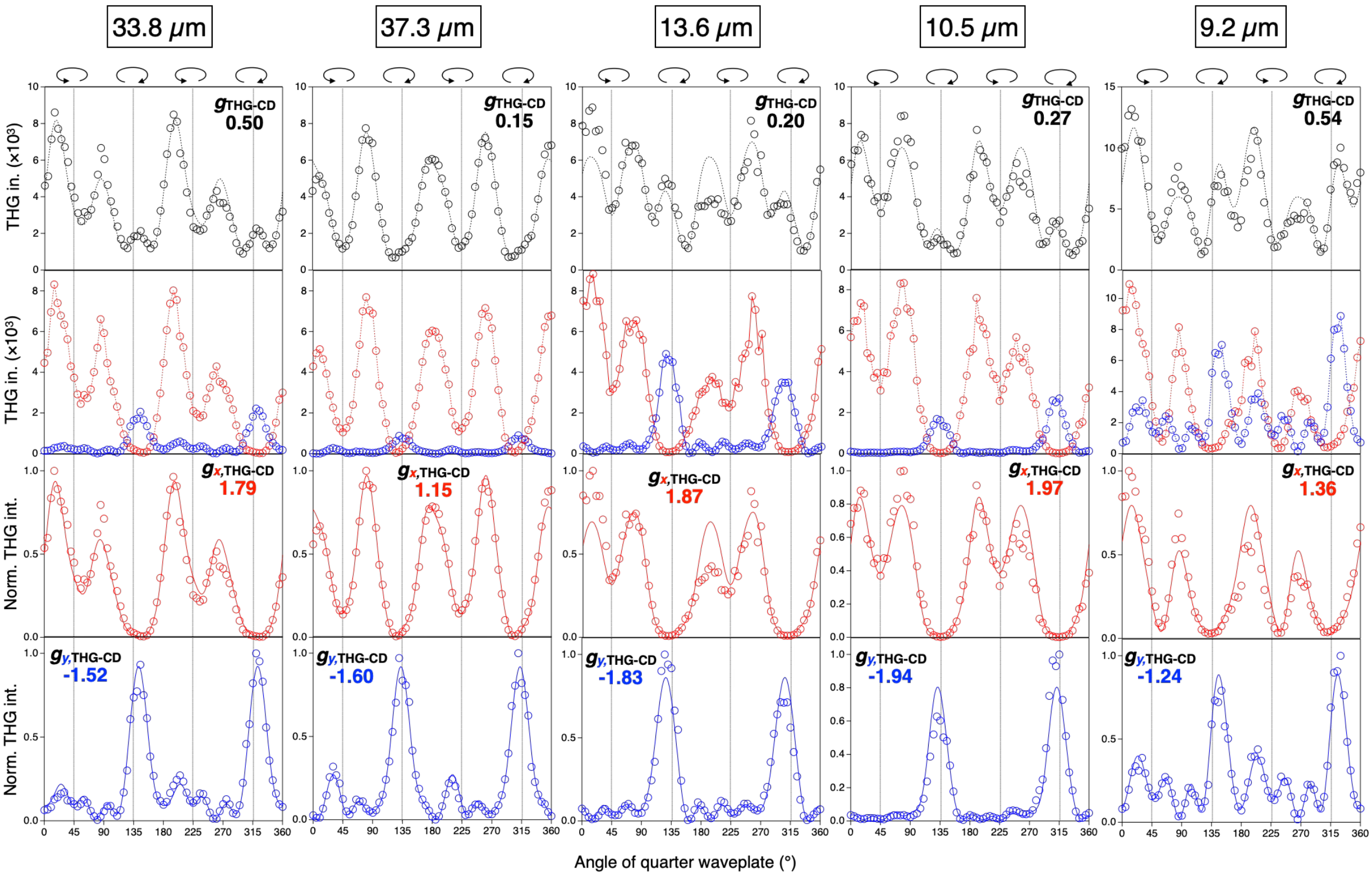

Figure S4. Total-THG intensity detected without polarizer (black) and x- (red) and y-polarized (blue) THG intensity profile as function of QWP rotation angle for R-SC sample with different thicknesses. The measurement was conducted under 1500 nm irradiations. The solid line shows fitting curve evaluated by Eq. 4.

8. Linear polarization degree of THG under the CPL irradiation in Figure 4a, b

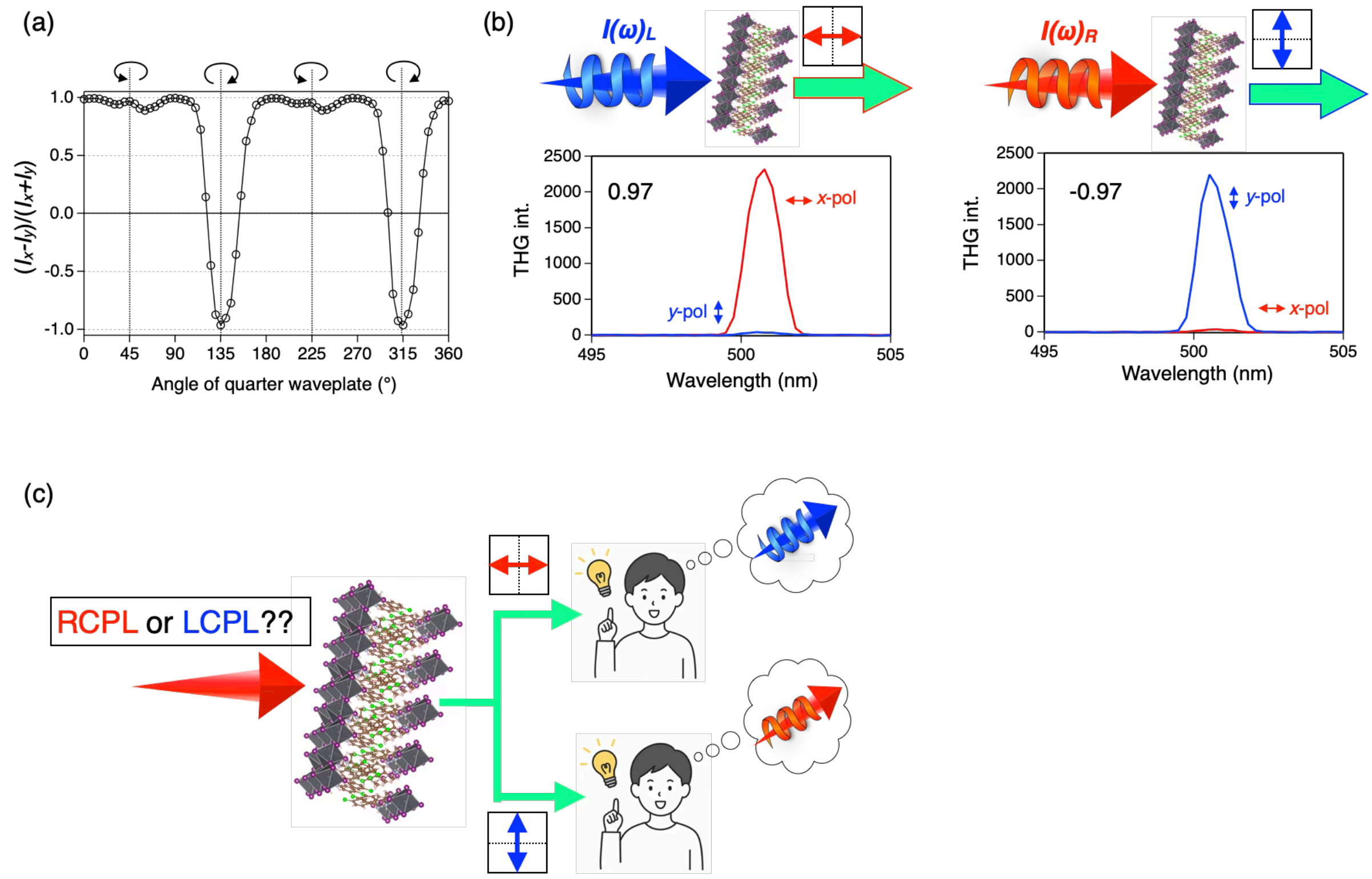

Figure S5. (a) Evaluated linear polarization degree as S_1 value of stokes parameter as function of QWP angle. (b) Extracted normalized *x*- and *y*-polarized THG spectrum under left and right CPL irradiation. (c) Schematic representation of a system for retrieving the incident circular polarization state from the output linear polarization.

9. Evaluation of stokes parameter of SHG under circularly polarized irradiation

In order to more accurately characterize the SHG polarization states, we evaluated all three Stokes parameters (S_1, S_2, and S_3) by using one of the crystals measured in Figure S4 showing good polarization resolved THG-CD (Figure S6a). The obtained normalized spectrum under each polarization state and the value are shown in Figure S6b, c. The degrees of polarization (DoP), calculated as $\sqrt{S_1^2+S_2^2+S_3^2}$, were approximately ~1, indicating nearly fully polarized SHG emission. The slight deviations of the evaluated DoP from 1 can be attributed to intensity fluctuations of the pulsed fundamental laser during the measurements. In addition, almost linear polarization with slight ellipticity was confirmed. The polarization azimuths differed by approximately 93°, demonstrating that nearly orthogonal polarization states were generated under L- and R-CPL irradiation, respectively.

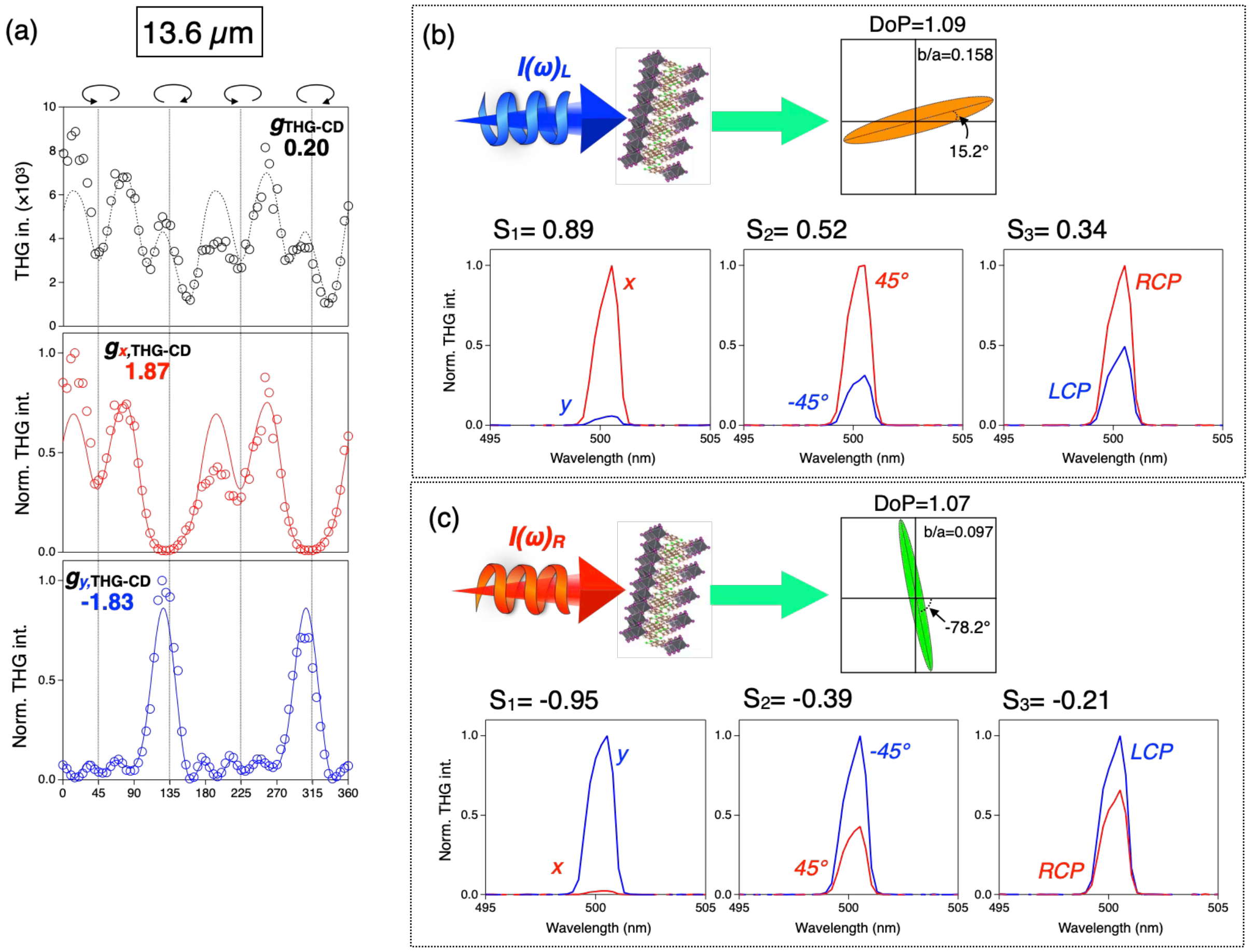

Figure S6. (a) THG intensity profiles as function of QWP rotation angle of the sample used for Stokes parameter analysis. (b, c) THG spectra measured at different polarization state with the evaluated Stokes parameters and corresponding polarization states under L-CP (b) and R-CP (c) fundamental laser irradiation.

10. Polarization resolved SHG-CD profile in S-SC sample showing weak $g_{\text{THG-CD}}$.

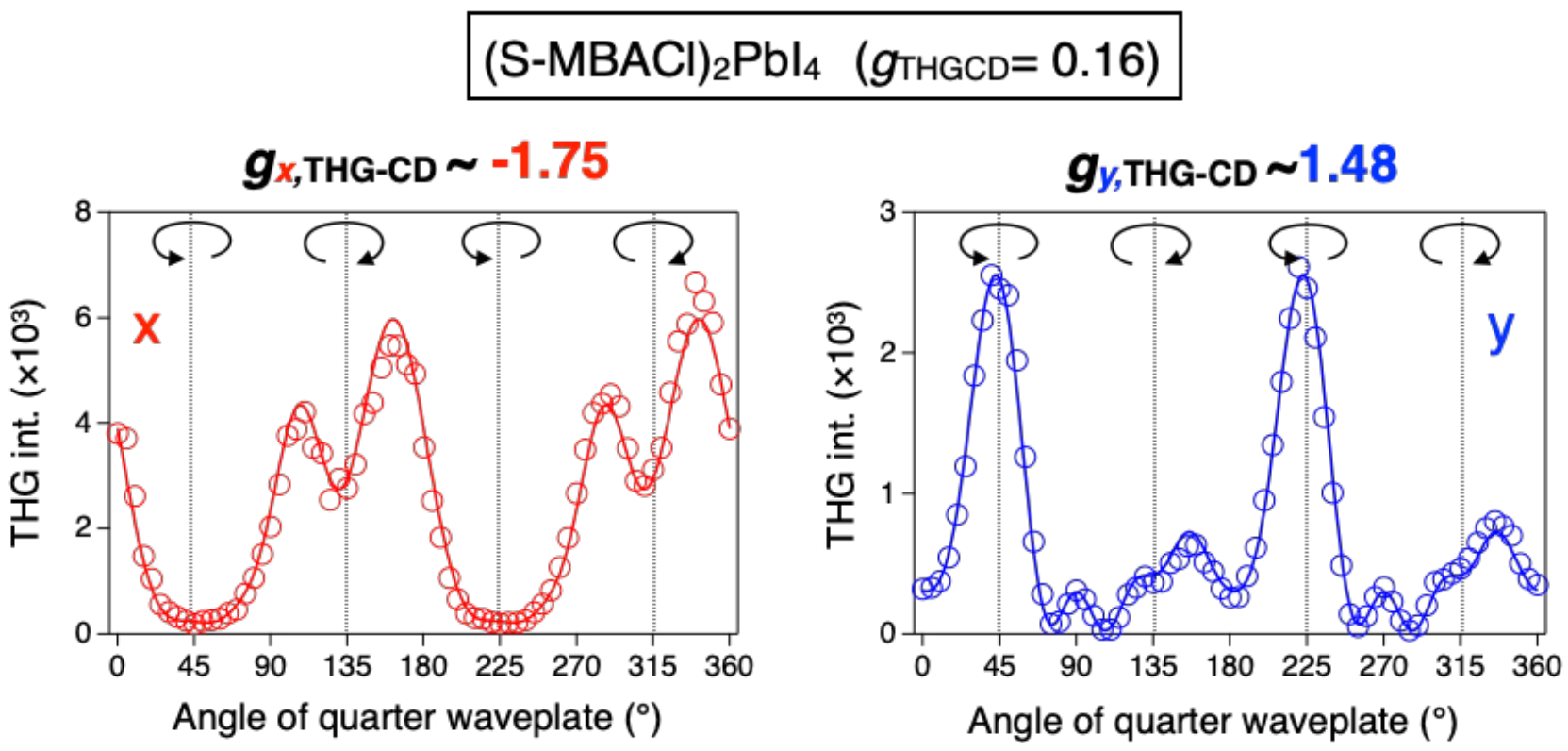

Figure S7. Polarization-resolved THG intensity as function of QWP angle in S-SC crystal.

11. Wavelength dependency of $g_{\mathrm{x,THG-CD}}$ in R- and S-SC sample

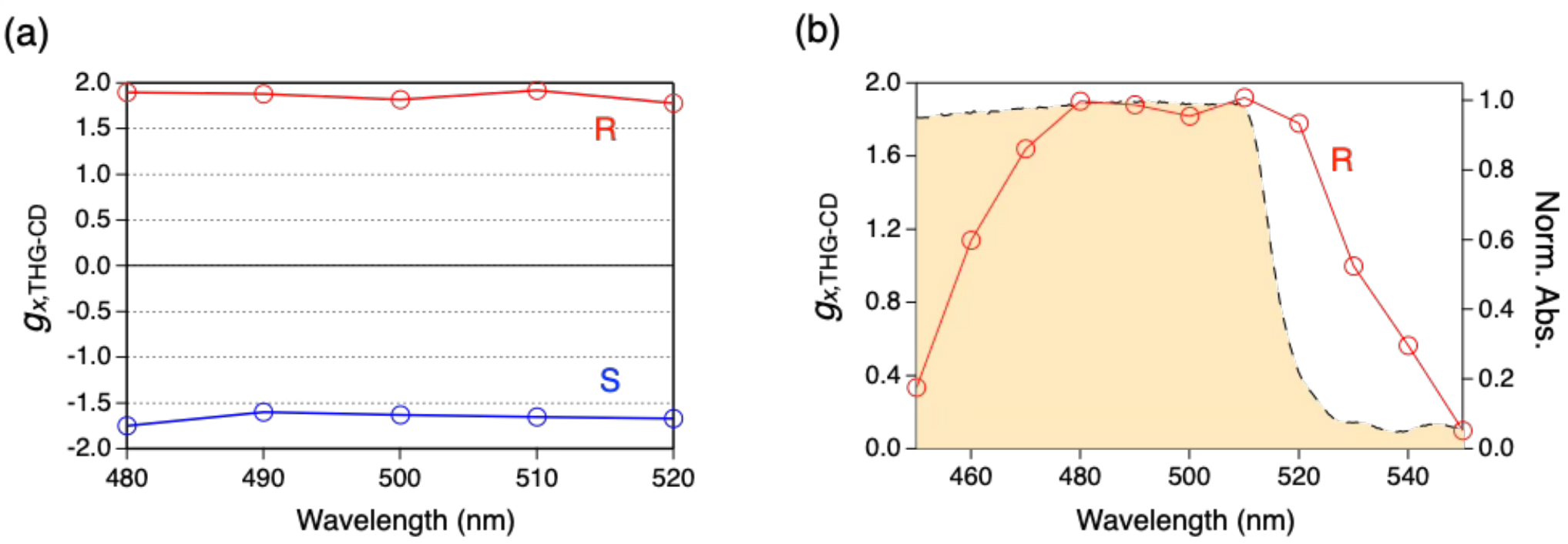

Figure S8. (a) Wavelength dependency of $g_{\mathrm{x,THG-CD}}$ in R- and S-SC sample. (b) Wavelength dependency of $g_{\mathrm{x,THG-CD}}$ in R-SC for wider THG wavelength range and normalized absorption spectrum in SC. The large $g_{\mathrm{x,THG\text{-}CD}}$ value is only observed near the excitonic resonance wavelength. These measurements were conducted using the same sample as that used for the data presented in Figures 4a, b, and S7.

12. Chiral THG response in achiral 2D perovskite crystal $(PEA)_2PbI_4$

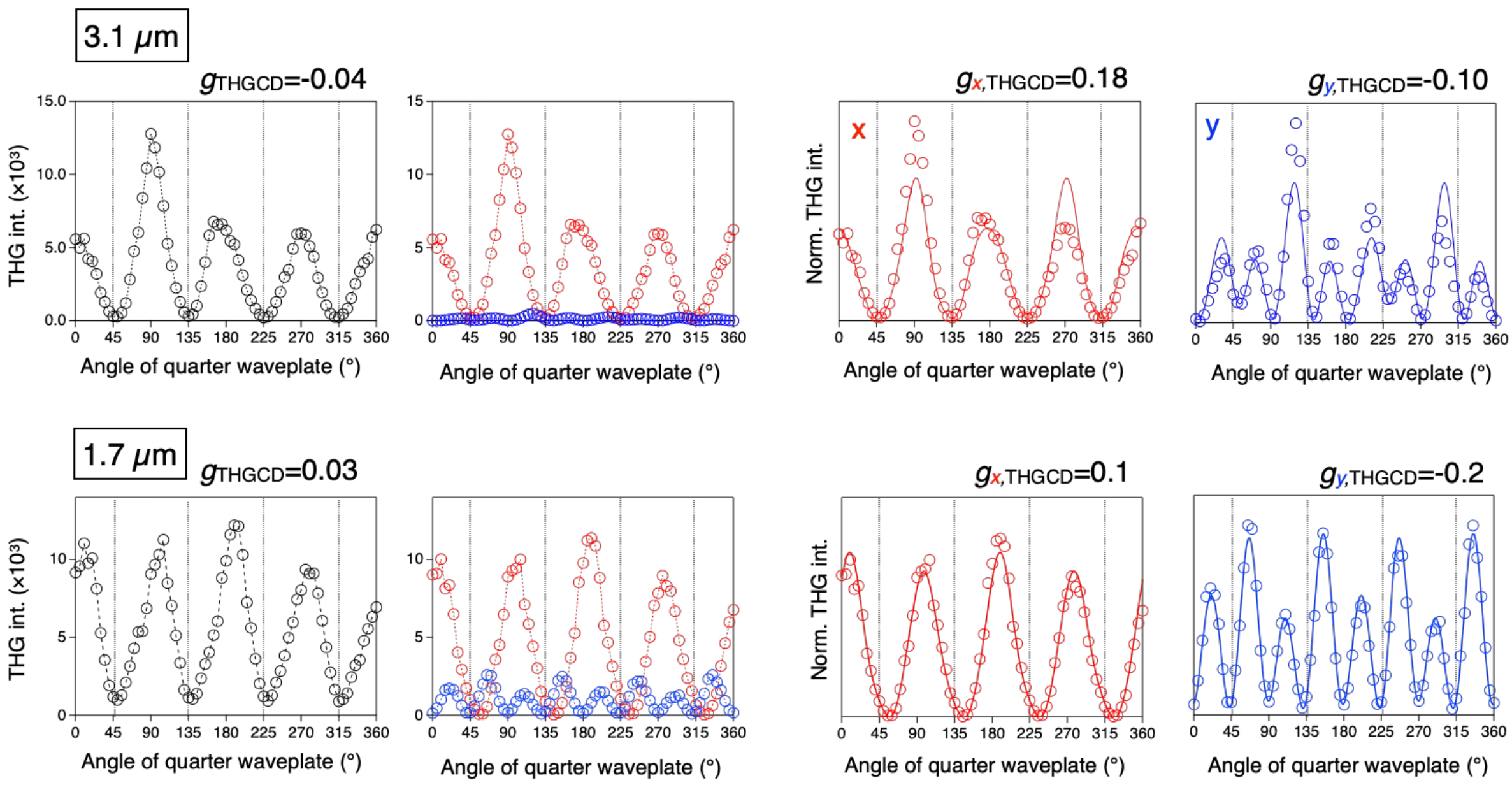

Figure S9. Polarization-resolved THG intensity as function of QWP angle in achiral 2D perovskite crystal with different thickness. The left black dot data show total THG intensity profile and red and blue data show *x*- and *y*-polarization data, respectively. The solid line overlapped with normalized *x*- and *y*-polarization data is fitting curve obtained using Eq. 4.

13. Hidden giant THG-CD response observed in different 2D chiral lead halide perovskite

Here, we also prepared and investigated chiral lead halide 2D perovskites with different chiral crystal structures: $(R\text{-}MBA)_2PbI_4$ (MBA: methylbenzylamine), belonging to the $P2_12_12_1$ space group, and $(R\text{-}3BrMBA)_2PbI_4$ (3BrMBA: 1-(3-bromophenyl)ethylamine), belonging to the $P2_1$ space group. Similar hidden chiral optical responses were observed in both crystals, indicating that this behavior is not specific to $(R/S\text{-}MBACl)_2PbI_4$ or its $P1$ crystal structure.

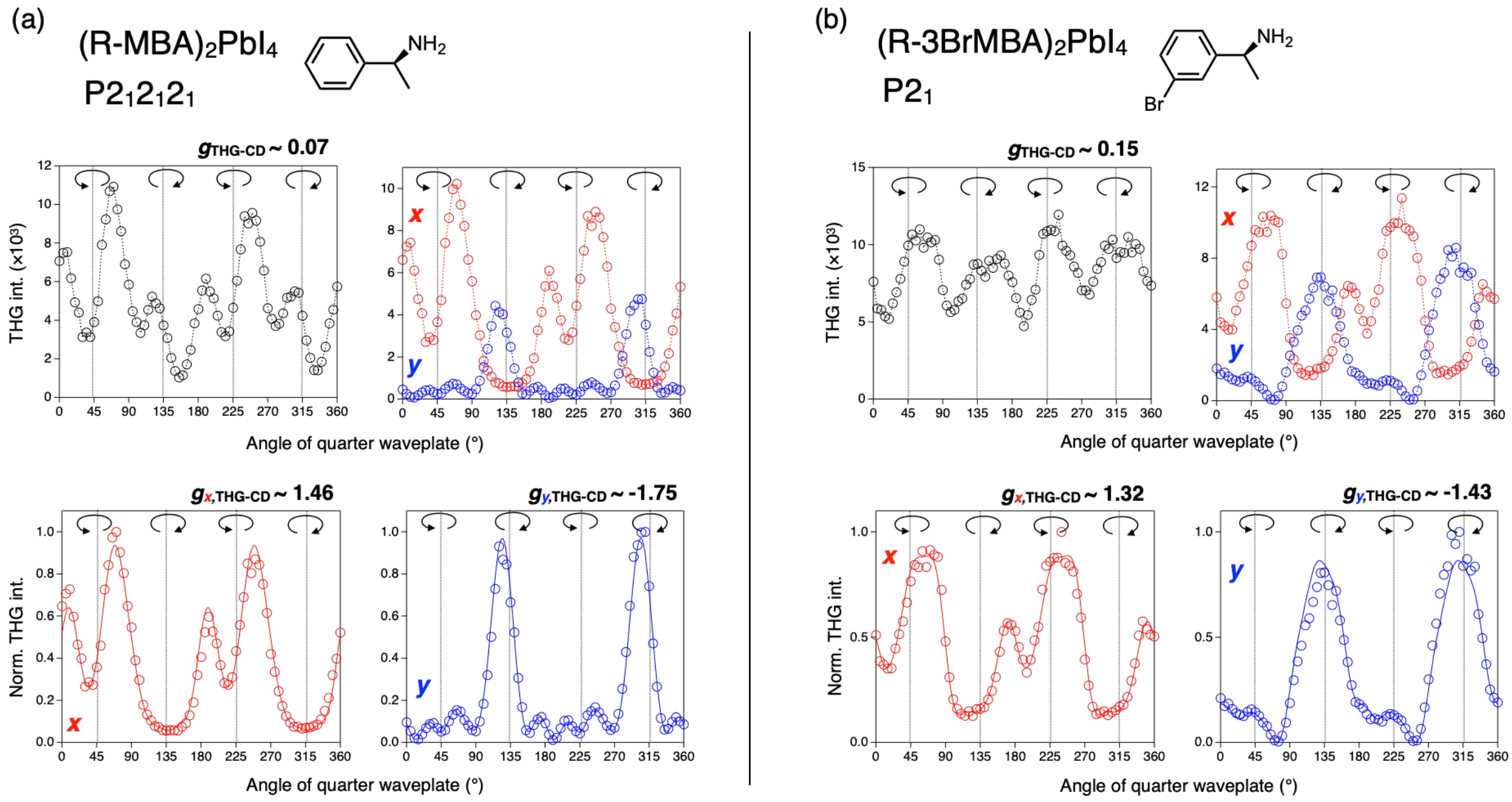

Figure S10. (a, b) THG intensity as function of QWP rotational angle in $(R\text{-}MBA)_2PbI_4$ (a) and $(R\text{-}3BrMBA)_2PbI_4$ single crystal.

14. THG-CD in $(R/S\text{-}MBACl)_2PbI_4$ thin film

THG-CD analysis for thin film

In the case of thin films, the samples are polycrystalline. Therefore, the crystalline orientation is assumed to be isotropically distributed, and the NLO susceptibility tensor can be approximated by an isotropic form:

$$\begin{pmatrix} P_X^{(3\omega)} \\ P_Y^{(3\omega)} \end{pmatrix} = \frac{\epsilon_0}{4} \begin{pmatrix} \chi_{11} & 0 & \chi_{11} & 0 \\ 0 & \chi_{11} & 0 & \chi_{11} \end{pmatrix} \begin{pmatrix} E_X^3 \\ E_Y^3 \\ E_X E_Y^2 \\ E_X^2 E_Y \end{pmatrix}$$

Considering CPL irradiation ($E_y = \pm i E_X$), both $P_X^{(3\omega)}$ and $P_Y^{(3\omega)}$ become zero. This indicates that both THG and THG-CD is not expected in the isotropic case under CPL irradiation. However, as shown in Fig. S11, clear THG-CD responses are experimentally observed in the thin-film samples. Unlike the single crystals, the thin films show the same sign of the chiral response in both polarization channels.

In the poly-crystalline thin-film samples, the small crystal domains are randomly oriented, not perfectly isotropic state, thus we can confirm finite THG activity even under CPL irradiation. In contrast, the polarization-dependent tensor contributions responsible for the sign reversal of the THG-CD response in single crystals are expected to be largely canceled through orientational averaging arising from the random orientation of crystallites.

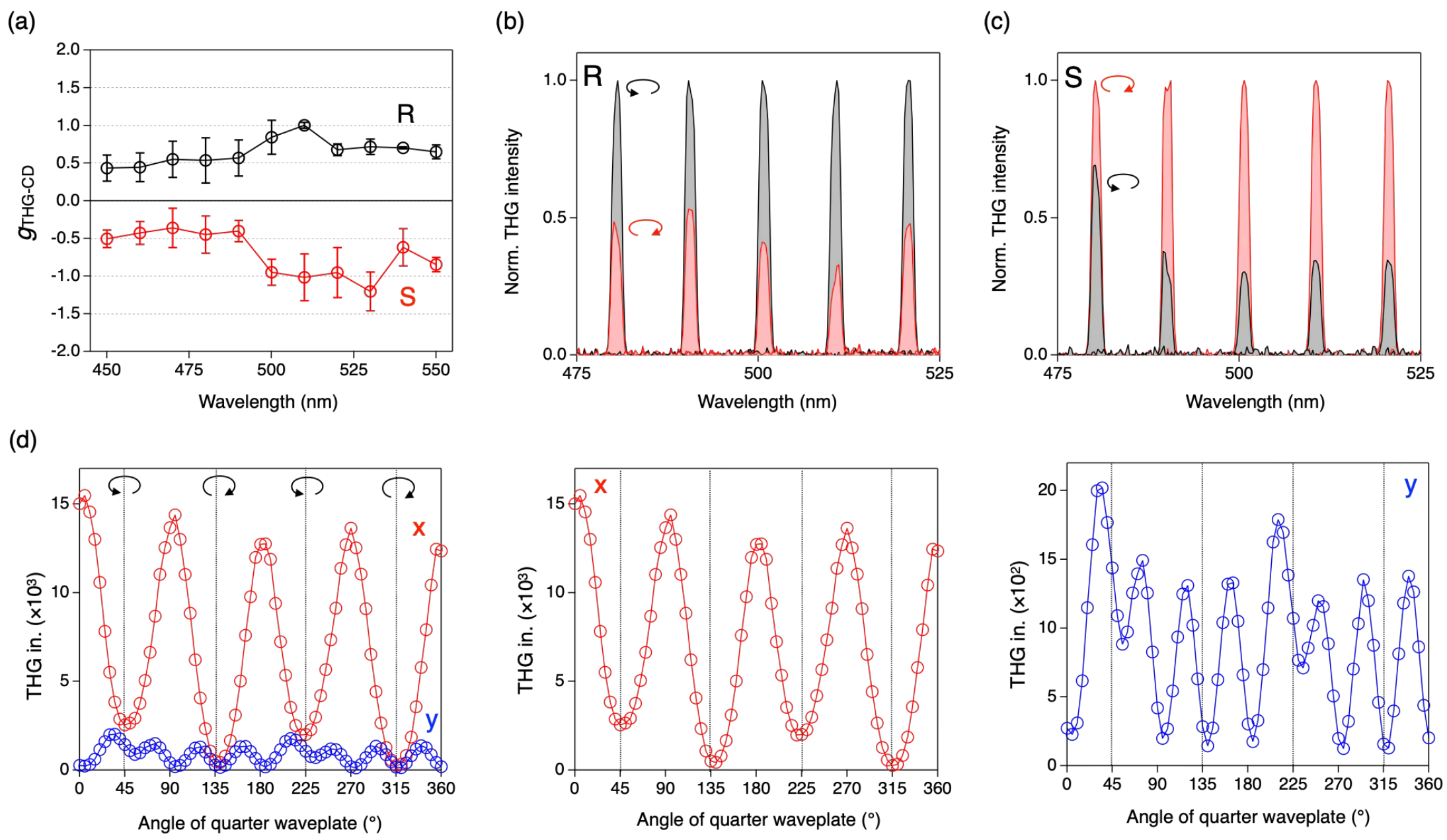

Figure S11. (a, b, c) wavelength dependency of g-factor (a) and normalized THG spectrum showing anisotropic response for R- (b) and S-TF samples (c). (d) Polarization resolved THG intensity profiles as function of QWP angles
